# The Hospital for the Insane

**Published:** 1890-03-22

**Authors:** 


### The Hospital for the Insane.

The able report of the Special Committee appointed by the London County Council to inquire into and to report to the Council upon the advantages which might be expected from the establishment, as a complement to the existing asylum system, of a hospital with a visiting medical staff for the study and curative treatment of insanity, met with scant courtesy at the hands of the members on the 11th instant. The Committee included Mr. Brudenell-Carter, who originated it and acted as its chairman, Mr. Longstaffe, M.B., F.R.C.P., Mr. Carr-Gomm, late chairman of the committee of the London Hospital, Captain James, R.E., chairman of the Sanitary and General Purposes Committee of the Council, and Mr. Martineau, chairman of the Asylums Committee of the Council.

This Committee, after taking the evidence "not only of experts in insanity and physicians chiefly engaged in the treatment of diseases of the nervous system, with which insanity is not necessarily associated, and also physicians and surgeons in more general practice," unanimously reported in favour of the establishment of a hospital for the insane. The following are the unanimous findings of the Committee :—

(*a*) That, in the opinion of the most eminent and most experienced members of the medical profession, the knowledge which is possessed, with regard to the nature, prevention, and cure of the diseased changes which underlie and occasion insanity, is not commensurate with that which is possessed with regard to diseased changes of other kinds, even those which affect other portions and other functions of the nervous system. (*b*) That the difference in question is mainly due to the circumstance that patients suffering from insanity have been to a great extent withdrawn from the operation of the ordinary methods of hospital investigation and treatment which have been so fruitful of good in the case of diseases of other kinds (*c*) That the establishment, on the ordinary lines, of a hospital for the study and treatment of insanity, with a visiting medical and surgical staff, could scarcely fail to be productive of increased knowledge of the subject and consequently, of increased means of prevention and of cure. (*d*) That the legal disabilities of the insane, and the necessity of subjecting them to a certain amount of restraint, render it impossible for the suggested hospital to be established by private benevolence, or by any other authority than that to which the care and treatment of the insane are committed by law.

The above recommendations constitute a fairly accurate summary of the report, and Mr. Brudenell-Carter, who presented it to the Council, moved the following resolutions:

(1) That an adequately-equipped hospital, containing 100 beds for the study and curative treatment of insanity in pauper lunatics of both sexes, be established in the metropolis, and that it be under the direction and control of the Council.

(2) That the staff and general organization of the hospital should be in accordance with the principles laid down in the preceding report.

(3) That a committee (which should be a sub-committee of the Asylums Committee) be appointed in order to carry the foregoing recommendations into effect, and to report their proceedings to the Council from time to time as may be required.

It was then moved by Mr. Cooper, seconded by Dr. Bott, and ultimately, after the application of the closure, decided :—

That the consideration of the report of a hospital for the insane be adjourned for six months, and that it be referred to the Asylums Committee for consideration and report.

Lord Rosebery thereupon stated that the terms of the resolution of the Council were not to be understood in a Parliamentary sense. The Council wished for six months' delay to decide as to whether this was a metropolitan or an Imperial question in the first place, and in the second place, granting it was a metropolitan question, whether they were justified, at a moment when the rates were acknowledged to be high and they had vast and new responsibilities to discharge, in adding this fresh burden upon the people.

We feel it is unfortunate that by a vote of something like 60 to 10 the London County Council should have thrown out Mr. Carter's resolutions; but we rely upon the assurance of Lord Rosebery that Mr. Cooper's amendment only involves six months' delay, when the whole question will be reconsidered on its merits.

